# General anesthesia for oral and dental care in paediatric patients with special needs: A systematic review

**DOI:** 10.4317/jced.57852

**Published:** 2021-03-01

**Authors:** Ana López-Velasco, Miguel Puche-Torres, Francisco J. Carrera-Hueso, Francisco-Javier Silvestre

**Affiliations:** 1 DDS. Service of Stomatology, Centro de Especialidades El Grao, Departamento Hospital clínico/Malvarrosa, Valencia; Pediatric dentistry Associate Professor Universidad European de Valencia, Valencia, Spain. Attached Dentist Center Specialties and Clinical/Malvarrosa University Hospital of Valencia. Assistant Professor Department of Pediatric Dentistry European University of Valencia, Valencia, Spain; 2 MD, PhD. Department of Maxillofacial Surgery, Hospital Clínico Universitario de Valencia, INCLIVA. Associate Professor, Surgery Department, Facultad de Medicina y Odontología Universidad de Valencia, Valencia, Spain. Scopus Author ID: 23477898100. Department Head maxillofacial surgery service, Clinical/Malvarrosa University Hospital of Valencia. Associate Professor, Surgery Department, Faculty of Medicine and Dentistry University of Valencia, Valencia, Spain 3 PharmD. PhD. Pharmacy Service. Hospital Universitario La Plana, Villa-real (Castellón) Spain. Attached Pharmacist University La Plana Hospital of Castellón, Spain; 3 PharmD. PhD. Pharmacy Service. Hospital Universitario La Plana, Villa-real (Castellón) Spain. Attached Pharmacist University La Plana Hospital of Castellón, Spain; 4 MD, DDS, PhD. Stomatology Unit. Hospital Universitario Dr. Peset de Valencia y Departamento de Estomatología Universidad from Valencia, Valencia, Spain. Attached Stomatologist Hospital Dr. Peset of Valencia. Professor Special Patiens Faculty of Medicine and Dentistry University of Valencia, Valencia, Spain

## Abstract

**Background:**

The objective of this study is to conduct a systematic review of the literature on the characteristics, needs and current situation of dental care for pediatric patients with special needs.

**Material and Methods:**

An exhaustive search for literature published until June 1, 2020. It was carried out using PubMed, Web of Science, Scopus, Cochrane and EBSCO, with the following keywords: Oral Surgical Procedures and Dentistry, Operational and Anesthesia, General Y (Spanish[lang] or English[lang] ) Y (infant[MeSH] Or child[MeSH] Or adolescent[MeSH]). The research was carried out following the PRISMA research methodology.

**Results:**

The most common indication for general anesthesia (GA) was tooth decay in 16 studies (6.5-90.8% of patients), followed by lack of cooperation and/or fear of dental professionals performing dental procedures in 8 studies. There is a higher prevalence of treatment in the group of patients with special needs, reaching 87.7% compared to 69.9% in healthy patients.

**Conclusions:**

In paediatric patients with special needs the use of GA is increasing, monitoring and preventive care are insufficient and withdrawal rates are high.

** Key words:**Oral surgical procedures and dentistry, operational and anesthesia, general.

## Introduction

Children with special needs are defined by having any physical, developmental, mental, sensory, behavioral, cognitive or emotional disabilities that require differentiated medical treatment, special medical intervention, and/or use of specialized services or programs. This definition can be applied in dental care, when due to the above characteristics these children require the use of appropriate behavioral guidance techniques, conscious sedation or general anesthesia (1).

Pediatric dentists provide dental care to children and adolescents who use non-pharmacological behavioral guidance techniques. However, to treat children with extensive dental problems, preschoolers, patients with physical or mental disabilities, patients who are medically engaged, who have general behavioral management issues, or who require maxillofacial surgery, dentists will need to supplement their oral treatment with pharmacological techniques, nitrous oxide sedation or GA.

GA is an efficient and safe resource for patients whose special characteristics make it impossible for treatment to be performed under local anesthesia or conscious sedation. Health services and treatment policies with respect to the General Assembly vary from country to country (2-8). We have observed that the use of GA is increasing in this patient profile, as preschoolers under the age of six and/or with mental disabilities lack the psychological maturity needed to tolerate dental treatment. Particular attention should therefore be paid to oral health promotion and education, as well as early prevention in pregnant women and to risk groups such as disabled patients (9-11).

Similarly, despite an overall decrease in the prevalence of tooth decay (12) and advances in preventive dentistry, restorative treatment and dental extractions are on the rise in this group of patients with special needs compared to healthy subjects of similar age, especially in the group of the mentally disabled (5,10,13). If we want to offer better quality of care, it is necessary to have adequate dental treatment under GA to improve the efficacy and safety of treatment and establish best clinical practices. This requires careful analysis of clinical evidence in order to provide adequate support for these children, taking great care to avoid further withdrawal as much as possible (4,5,9,10,14-16).

The main objective of this systematic review is to determine the characteristics of care for children who are medically engaged, have significant disabilities or behavioral difficulties, who undergo GA for oral health care procedures.

## Material and Methods

A structured literature search was conducted using the databases PubMed, Web of Science, Scopus, Cochrane and EBSCO, with the following keywords in Medline/PubMed: Oral Surgical Procedures and Dentistry, Operational and Anesthesia, General Y (Spanish(lang) or English(lang)) Y (infant(MeSH) O child(MeSH) or adolescent(MeSH)) in various combinations. The search covered all published articles with no time limit. A search of grey literature was carried out in the doctoral thesis databases, as well as a manual review of the literature included in the articles.

The inclusion criteria were full-text articles, regardless of study time or year of publication, until 1 June 2020. We include original articles published in scientific journals in English and Spanish; prospective and retrospective observational analytical studies and literature reviews, specifying oral dental treatment under general anesthesia in children up to 18 years of age. No restrictions were applied in terms of population classification or diagnostic criteria. The control group (CG) was the healthy patient population.

**Figure 1:**
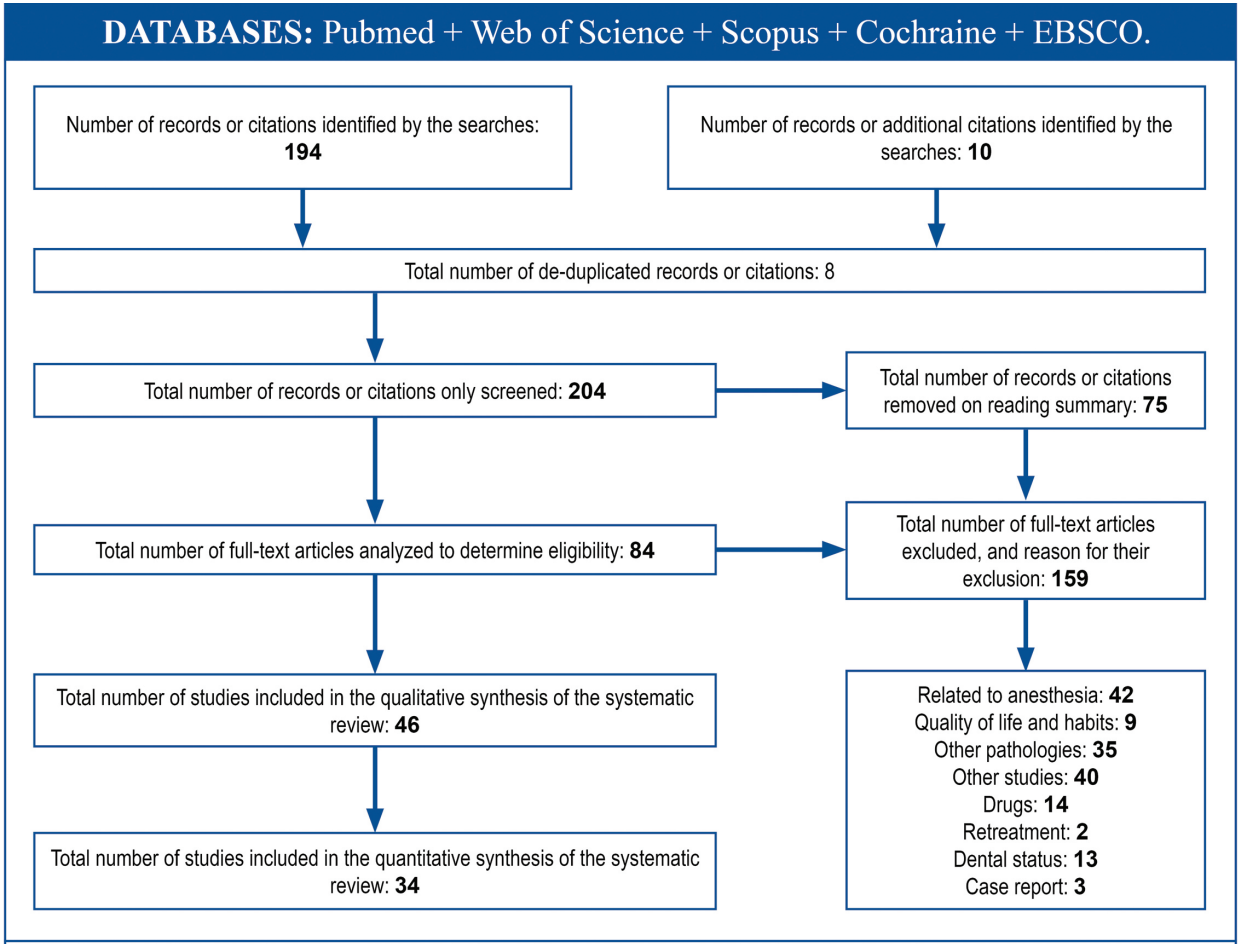
Flow of information through the different phases of systematic review.

Studies of articles related to any type of analgesia, or behavior (management programs, oral health habits...), case reports or other non-GA studies, as shown in the flowchart (Fig. 1) are excluded from our review.

A structured shape was used to definitively extract and collect data from studies selected by two independent authors (ALV and FJCH). The discrepancies when comparing the results of the two authors were resolved by a third party (MPT). To evaluate the quality of the selected studies, each of them was scored according to the Strobe scale by two researchers (ALV and FJCH). Disagreements were resolved by consensus with a third investigator (MPT). The average score obtained in each study with a cut-off point of 14 was used to define accepTable quality.

The main variables collected from each study were the design and characteristics of the study, the health status of patients according to the classification of the American Society of Anesthesiologists (ASA) (17), pathology or procedure indicated for surgery, pending surgery, interventions performed, post-surgical complications, results and follow-up time. In comparative observational studies, clinical results were taken separately from both groups of patients studied.

Clinical treatment results were evaluated using two parameters: A) results expressed in percentages; B) average treatment per tooth and child. If any article expressed the results in a different way, this was included and clarified accordingly. We evaluated the retreat over time in studies targeting this area and finally assessed whether prevention had been carried out.

Ethical Approval: This article does not contain any studies with human or animal participants conducted by any of the authors.

## Results

1. Search results

A total of 204 articles were obtained, of which only 34 studies met the inclusion criteria. After removing 8 duplicates, a total of 196 items were obtained for analysis. Articles related to drugs or analgesic/anesthetic techniques, clinical cases related to a specific pathology and other non-GA studies were eliminated, applying exclusion criteria after reading the summary, or if the full document is necessary.

Finally, 34 publications remained for analysis. The stroBE scale interobserver evaluation of the included articles showed great homogeneity among researchers (kappa>0.78).

2. Features of the study

The main characteristics of the selected studies are shown in Table 1,  Table 1 cont.,  Table 1 cont.-1. All selected studies were cross-cutting, published between 1967 and 2017. The number of patients studied was highly variable, ranging from 40 to 1000 patients; only one study, in South Africa, was higher than this range, with 16732 patients. In fifteen studies the treatment was evaluated by means of averages and three by percentages. Eight studies presented their results in percentages and means, and three studies did not evaluate treatment (Table 2). We have considered studies in children up to 18 years of age: six studies did not meet this criterion, but the average age was for children between 10 and 17 years old. The genre was not specified in most published articles.

**Table 1:**
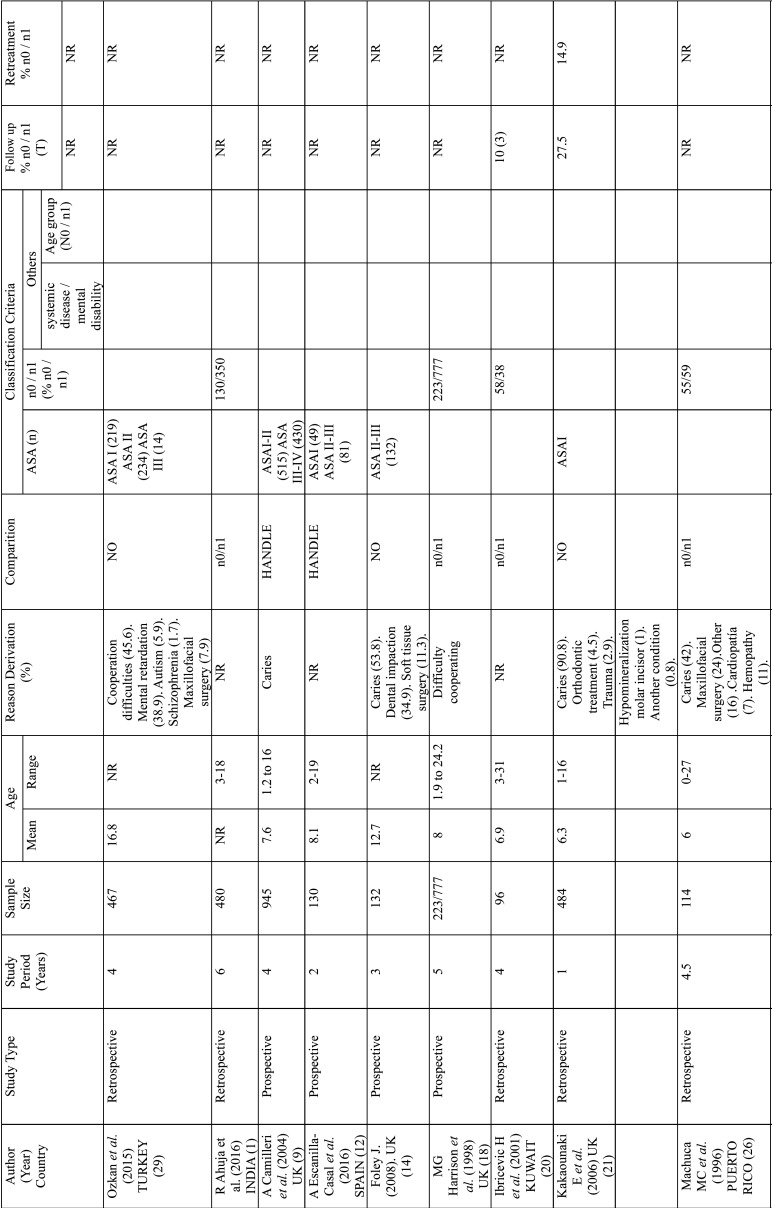
Main characteristics of the studies included.

**Table 1 cont.**
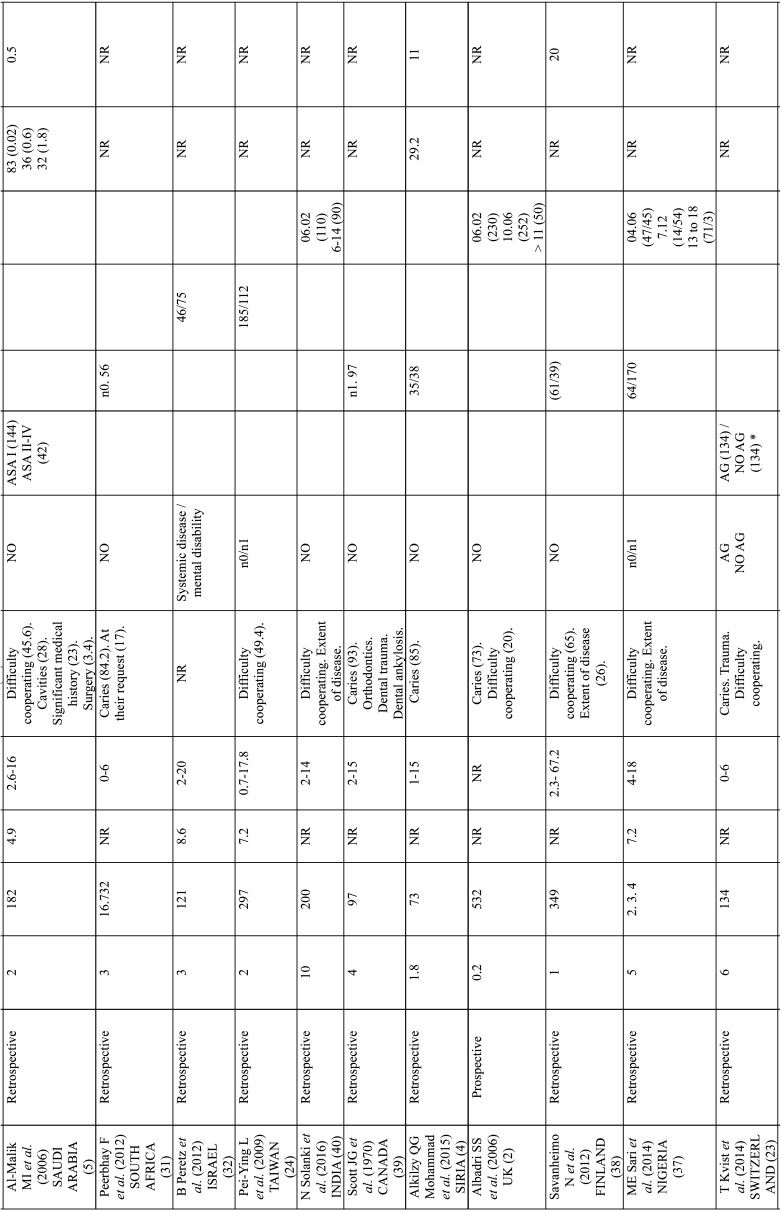
Main characteristics of the studies included.

**Table 1 cont.-1:**
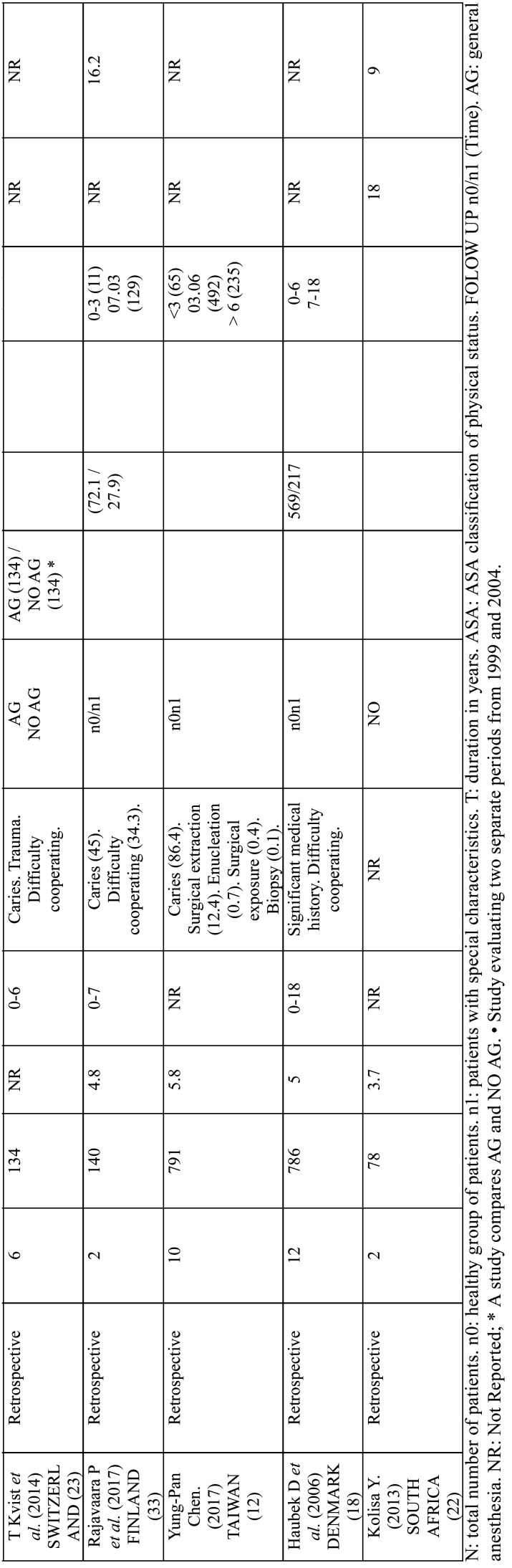
Main characteristics of the studies included.

**Table 2:**
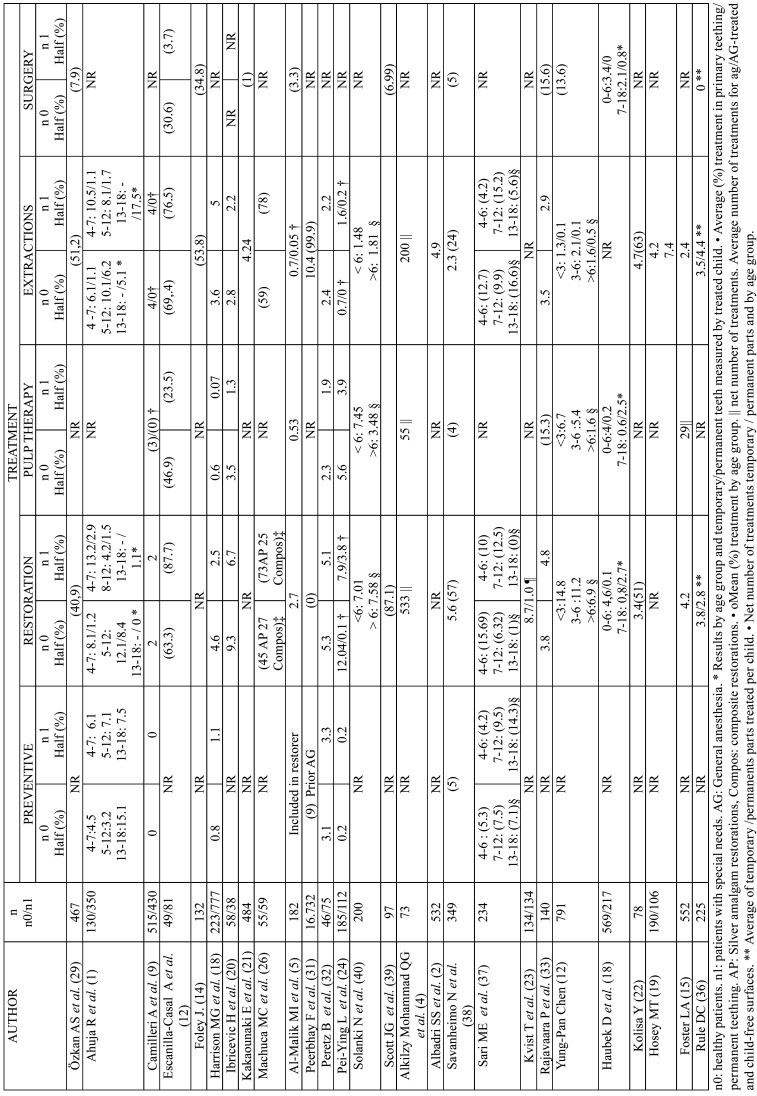
Results by treatment type.

The studies analyzed mark a very varied follow-up period of between 1 and 13 years.

Tooth decay was the most common cause of reference, in seventeen studies (6.5-90.8% of patients), followed by lack of collaboration/dentists, eight studies (5.2-45.6%). Other reasons were intellectual disability in Foley’s study (34.9%) and dental impact. Five of the studies did not specify the reason.

Some studies did not identify common demographic variables. In some studies, no demographic variables such as gender were identified, or the study group was defined differently, dividing patients, for example, by age group. In some cases ASA classification is used, while in others the study group was defined as patients with developmental disorders, special patients or with mental disabilities. Fifteen studies lacked a control group and one study compared patients who underwent GA with those who did not (31).

Patient follow-up was rarely recorded, ranging from 10% to three years after the General Assembly procedure (9), 27.5% in periodic preventive follow-up visits (4), and 83% per week after intervention (21).

The Alcaino E. study evaluates treatments in two periods of time, in ten years these increased by 150%. The number of children treated under GA in the study of P. Rajavaara also increases by 50% in just one year.

Most of the studios were a single center. Only one was multicenter with the involvement of two hospitals. In terms of time lapse, 25 studies were retrospective, 8 prospective and 1 was ambispective.

We found great heterogeneity among the studies on methodology. Although most studies were based on treatment, five studies reported between 0.5 and 16% recurrences after GA (4,15,21,25,28).

The main findings regarding treatment evaluation are shown in Table 2, with results expressed in percentages. Eleven studies evaluated the results in percentages, only three of them with control group, with the following variations: restoration procedures 0-87.1%; pulp therapy procedures 7-40.9%; dental extractions: 14.9-99.9%; surgical procedures: 1-46.2%; preventive treatment: 4.2-20.5%.

In studies comparing special needs with healthy patients, there was a higher prevalence of restorative, preventive and exdondontic treatment in the group of patients with special needs.

In fifteen studies (five with comparator) the results were also expressed in means of treatment per tooth and child: restoration procedures: between 0.1 and 14.8; Pulp therapy procedures: 0.02-7.45; dental extractions: 0-17.5; surgical procedures: uns specified; preventive treatment: between 0 and 7.5.

Five’s studies distinguish between results in temporal and permanent teething (14,15,25,34,35), three of which included a control group; the results were that the prevalence of treatment is higher in temporary teething and in the group of healthy patients.

Six other studies, Ahuja, Solanski, Sari, Haubek, Rule and YP Chen divide patients by age group. Two of them compare special needs against healthy patients, reporting a higher prevalence of treatment in the special needs group and in the group of patients over 6 years of age. Differentiation by type of treatment, in children under 6 Six other studies, Ahuja, Solanski, Sari, Haubek, Rule and YP Chen divide patients by age group. Two of them compare special needs against healthy patients, reporting a higher prevalence of treatment in the special needs group and in the group of patients over 6 years of age. Differentiation by type of treatment, in children under 6 years restoration treatments are most often carried out in healthy patients, with the exception of dental extractions that are most common in children with special needs.

Kvist and others compare children under the age of 6 treated with and without GA, with the average number of restorations seven times higher in children receiving GA. Dental neglect and dental disability were found significantly more often in children treated with GA (32,33).

It should be noted that only eight studies evaluate periodontal treatment and nine evaluate preventive treatment; this is not done systematically in all children, although most authors refer to its importance in the prevention of dental pathology (9,10,31), which turns out to be the most common dental pathology, between 14.8 and 90.8% (4,27).

## Discussion

The use of GA in paediatric oral care procedures is increasing (9,27,32,33,37,39), both in healthy patients and in patients with special needs. Although the cost of this service can be high, comprehensive dental treatment is carried out in a single session and requires minimal cooperation on the part of the patient. Therefore, it is safe and efficient and in most cases, it represents the only way to provide dental treatment to these patients with special needs (9,15,23).

Withdrawal was most common in patients with special needs (15,25,28,33); Kakaunaki’s study was only one with most healthy patients reporting a similar percentage of repeated GA. In our view, the high proportion of GA recurrence, up to 16% in selected studies, is mostly due to new cavities in children with severe medical condition. These patients have a higher incidence of tooth decay due, among other factors, to the greater amount and particular consistency of carbohydrates present in the diet, sugar content in prescribed medications, lower salivary flow in the oral cavity and poor hygiene. Dental treatment with AG, in a single session, is the most appropriate and feasible option in these patients (9,10,23,28,34).

The pharmacological, medical, surgical and rehabilitation treatment received by patients with special needs for systemic pathology can affect oral and dental tissues, and this plays an important role in the planning of oral treatment; the dental treatment provided may also influence the efficiency of general treatment and the course of systemic disease (5,9,13,17).

Numerous studies suggest that the underlying medical or mental conditions of these patients may influence the dental condition and treatment modality provided. For this reason the treatment protocol sometimes needs to be modified, adopting a more aggressive dental treatment strategy, such as tooth extraction rather than conservative treatment (5,9,10,16).

It should not be forgotten that dental problems can place an additional burden on children with special health care needs, due to the additional hospitalization needed to treat a variety of medical conditions on more severe occasions (9,10,23,28,34). Our review found that 19 of the 34 studies looked at pulp therapy, which is carried out much less frequently and again to a lesser extent in the group of patients with special needs (7,9,13,22,25). The highest number of permanent dental extractions among subjects with disabilities may indicate that in this group of patients dentists prefer to permanently remove severely damaged teeth with questionable prognosis, rather than risk the need for retreat. Therefore, we agree with Harrison and Ibricevic *et al.* that in children with special needs certainty regarding the outcome of dental treatment is essential.

On the other hand, in studies comparing patients with special health care needs and healthy patients, there are generally a greater number of procedures in patients with special needs. According to studies conducted by Ibricevic, Tahmassebi, Sari, Barberia and Haubek, there are more restorative treatments in the group of healthy and young patients (10), in the latter case due to the prevalence of cavities in early childhood. In studies conducted by Machuca and Salles, restorative and exodontodic treatments predominate in the group of patients with special needs. In the Rajavaara GA study it increased by 50% in just one year and there were more restorations in patients with special needs, while in Peretz’s study there were no significant differences between the groups.

The distribution of patients treated with GA varies depending on the age group and underlying disease. As the age of the ratio increases, the need for GA treatment in healthy individuals is eliminated. On the other hand, the number of patients with intellectual disabilities and comorities treated with GA increases in line with age (9-11).

A patient may experience progression of oral disease if treatment is not provided due to age, behavior, inability to cooperate, disability, or medical condition. Deferral or denial of dental care can result in unnecessary pain, increased treatment needs and costs, and ultimately a more acute quality of life.

All of this highlights the need to consider children with special needs a high priority group and to take into account the risks of developing oral diseases that require more intensive preventive care, further monitoring of treatments under the General Assembly, and continued promotion of oral health (25). After comprehensive dental care under GA, most healthy children can usually be treated in dental surgery under local anesthesia, but still require special preventive care and behavioral guidance, due to their lack of cooperation and fear of dental procedures. This will help reduce non-compliance with periodic controls (21,31). Only four studies record follow-up, ranging from 10 to 83% (4,9,21,25).

Indications for the application of GA should be based on specific criteria, including risks, benefits, efficacy, expected results and the use of other behavioral guidance techniques as an alternative. These patients require greater attention and additional effort in terms of oral and medical treatment, and pose a major challenge for the professionals involved (33).

## Conclusions

The use of GA for paediatric patients with special care needs is increasing, there is little monitoring of these patients and preventive care is insufficient, with high withdrawal rates.
